# Representation of shared surface information and false memory for abstract versus concrete pictures in the conjoint recognition paradigm

**DOI:** 10.1007/s00426-023-01899-5

**Published:** 2023-12-14

**Authors:** Marek Nieznański, Daria Ford, Michał Obidziński

**Affiliations:** Institute of Psychology, Cardinal Stefan Wyszyński University in Warsaw, ul. Wóycickiego 1/3 bud. 14, 01-938 Warsaw, Poland

## Abstract

An effective factor by which false memories can arise is relatedness which includes not only semantic associations but also perceptual resemblance. This issue raises questions about how patterns of perceptual features are represented in memory and how they relate to semantic representations. In five experiments, we investigated the memory processes underlying the false recognition of perceptually or semantically related pictures from the perspective of fuzzy trace theory. Multinomial processing tree model analyses for the conjoint recognition paradigm showed that the parameter representing gist trace retrieval not only contributes to false acceptances of semantically related pictures, but also underlies the false recognition of non-semantically related abstract shapes. These results challenged the hypothesis that the false recognition of non-semantically related distractors is solely due to interference with the verbatim suppression process. These experiments also showed that adding a surface feature (colour) to the category exemplars increases false recognition of related distractors by enhancing the contribution of the familiarity process, but only for pictures of real objects. Comparisons between experiments showed that different variants of the conjoint recognition model, used to analyse the effects of the same experimental manipulation, can lead to partially different conclusions.

## Introduction

False memories have received considerable interest in experimental psychology in recent decades (for review see: Brainerd & Reyna, 2005; Gallo, 2006). A powerful factor by which false memories can arise is relatedness. The presentation of related lists of items produces false memory for items not in the original lists but related to them (Roediger & McDermott, 2000). Most research in this field has been conducted using the well-known Deese/Roediger–McDermott (DRM) paradigm (Deese, 1959; Roediger & McDermott, 1995). In this task, participants study lists of words (e.g. *bed*, *rest*, *awake*, *dream,* etc.) semantically associated with a non-studied word (*sleep*), which is used as a critical distractor during a memory test. The usually observed high false acceptance rate of critical distractors was analysed from a variety of theoretical perspectives. In this article, we focus on the approach rooted in fuzzy trace theory (FTT), developed over the years by Brainerd, Reyna, and their co-workers (e.g. Brainerd & Reyna, 2002; Brainerd et al., 1999, 1995b; Reyna et al., 2016). This theory postulates that two types of memory traces are encoded in parallel: *verbatim traces* containing integrated, item-specific, surface information, and *gist traces*, storing the meaning content of experienced items. FTT suggests differences in the kind of memory processes on which accurate and false memories are based in DRM-like paradigms: verbatim and gist trace retrieval both support true memories, whereas gist trace retrieval supports false memories of related distractors, and verbatim trace retrieval suppresses false memories. For recognition memory, FTT assumes that targets presented during the test are generally better retrieval cues for verbatim traces than any of the distractors. In the case of semantically related distractors, they may cue retrieval of gist traces, leading to false alarms based on *familiarity*. Moreover, a strong gist trace retrieval can induce a memory illusion represented by a process called *phantom recollection*. Compared to familiarity, which is accompanied by feelings of global similarity between the distractor and the material studied, phantom recollection is accompanied by an illusory conscious experience of the details of the presentation of this distractor on the study list (Brainerd et al., 2001). Finally, the presentation of the related distractor may result in the recollection of the corresponding target’s verbatim trace and the judgement of non-identity of this distractor to the target. This process leads to a reduction in the incidence of false alarms and is called *recollection rejection* (for more details on FTT approach to memory see: e.g. Brainerd & Reyna, 2002; Brainerd et al., 2001; Nieznański & Obidziński, 2019; Nieznański et al., 2018, 2019; Reyna et al., 2016; Stahl & Klauer, 2008, 2009).

Based on FTT, Brainerd and colleagues (Brainerd et al., 2014, 2015) developed a dual-recollection theory that distinguishes between the conscious recollection of contextual information and the vivid reinstatement of target information. In this model, *target recollection* derives from the correct retrieval of verbatim traces of old items, and generates feelings of contrast between a remembered target and a distractor that resembles but mismatches the target. *Context recollection* is based on gist trace processing, and results in false acceptance of distractors that share features of targets because of the retrieval of contextual details that were associated with those targets’ presentations (Brainerd et al., 2014). Therefore, as in original FTT, the dual-recollection theory postulates that false memories stem from the gist processing of related distractors (familiarity or context recollection) or from a failure of verbatim processing of targets (interference with target recollection). The purpose of the current study was to investigate, from the above theoretical perspective, the processes underlying the false recognition of perceptually similar material and the consequences of adding a perceptual feature to elements of a class of concrete or abstract stimuli.

### Processes contributing to false recognition of perceptually similar distractors

For both FTT and dual-recollection theory, the question arises about processes contributing to false recognition of surface (perceptually) similar distractors. According to these theories, surface features are encoded into verbatim traces, which excludes gist-based processes of familiarity or phantom recollection (context recollection) as contributors to false recognition of perceptually (but not semantically) related distractors. In result, false recognitions of these distractors should be due to interference with the verbatim suppression process, that is, recollection rejection (target recollection) (Chang & Brainerd, 2021). Therefore, it is expected that verbatim trace retrieval failures are responsible for the ineffective rejection of perceptually similar distractors. Any factor that makes discriminating targets and distractors’ perceptual features harder should result in decreases in recollection rejection and, in consequence, a higher level of false memory than for easy discriminable items (cf. Nieznański & Obidziński, 2022). In this approach, it is clear that perceptual similarity leads to the failure of false acceptance *suppression*, however, it is unspecified how these false acceptances of related distractors as targets are *fomented* (if not by gist-based processes).

In our recent study, on false memory for orthographically (phonologically) related words (Nieznański et al., 2019), we found high levels of gist memory estimates for critical words orthographically related to studied words. Therefore, we proposed a redefinition of gist trace as based not only on shared meaning but also on orthographic patterns (cf. Holliday & Weekes, 2006). In this proposition, verbatim trace encodes integrated item-specific surface information, whereas gist trace encodes both semantic and surface commonalities (patterns) between items. This account differs from the well-documented assumptions of FTT and dual-recollection theory (Chang & Brainerd, 2021). However, it is possible that gist-trace contribution found in our experiments was in fact a product of the meaningful materials used for study. Perhaps, rhyming words, though not directly semantically related, activate each other’s meanings (Brainerd et al., 1995a) leading to gist trace contribution during a memory test. Therefore, in the current study, to reduce the possible confounding effects of semantic and surface features, we used abstract shapes with no pre-existing semantic meaning instead of words to study the more isolated effect of surface resemblance on false memory.

The first aim of this research was to test FTT and dual-recollection theory assumption that interference with the verbatim suppression process is the main source of false memory for non-semantically related distractors (Chang & Brainerd, 2021). However, as we mentioned earlier, this is rather a process aimed at reduction than fomenting false memory. If gist trace is purely semantically grounded, using abstract shapes instead of words should eliminate the contribution of gist trace retrieval to false acceptances. Familiarity would contribute to false acceptances only if we assume that it is not solely based on gist trace retrieval or gist is not solely based on shared meaning but also on perceptual patterns (or equivalently, there are two types of gist: perceptual and semantic). An interpretation of familiarity as reflecting processing fluency of items’ perceptual features is well-known from classic dual-process models of memory (Jacoby, 1991; Jacoby & Dallas, 1981; Mandler, 1980). However, a recent meta-analysis of conjoint-recognition studies (Brainerd et al., 2022a) clearly rejected an interpretation of familiarity as being grounded in the perceptual features of old items.

### The role of adding a surface feature to members of a category for false recognition

The second aim of the current research was to study the role of adding a surface detail to the targets belonging to a category, for false recognition of distractors belonging to this category and sharing this surface detail (cf. Nieznański & Obidziński, 2022). This issue is connected to our main research question about how shared surface details are represented in memory; the manipulation of adding a surface feature tests the consequences of strengthening relatedness within a category of stimuli and decreasing it between categories. If a gist retrieval drives false acceptances of related distractors, false recognitions are predicted to increase when an additional perceptual feature is shared by items, building a category represented in a gist-trace. The question remains whether such an effect occurs only for classes representing semantic content or also for abstract perceptual patterns.

A natural hypothesis stemming from FTT is that perceptual features are stored in the verbatim traces (Chen et al., 2018). However, the results of Chen et al.’s (2018; cf. Ball et al., 2014) experiments suggested that a more likely scenario is that surface details are stored with traces of semantic information (e.g. taxonomic information about list items). In other words, according to Chen et al. (2018) surface contextual details are stored with representations of semantic content. A similar issue was discussed in a paper by Brainerd et al. (2014), which noted that false alarms to a new word from a semantic category are often accompanied by recollection of criterial contextual details that were studied along with a block of words from that semantic category.

Following these suggestions, in our research, we predict that providing an additional surface detail (such as colour) that is shared by items belonging to one category, will help subjects to extract semantic information connected to this category and increase false recognition of distractors belonging to this category. Such an increase in false recognition will occur when targets and distractors belong to common semantic category, but not necessarily when the category is built on surface resemblance. Therefore, in the case of abstract shapes, for which semantic-gist representation is not encoded, the effect of providing category-specific perceptual feature is ambiguous. In contrast, this effect should be present for pictures belonging to concrete semantic categories since semantic familiarity contributes to false memory.

Most recently, Brainerd et al. (2022a) have considered an alternative possibility that contextual details are stored in a type of memory trace that is separate from verbatim and gist, namely, a contextual trace. Contextual details are distinct from surface and semantic details specific to particular items since they are associated with multiple studied items. The three-dimensional structure was confirmed in Brainerd et al.'s (2022a) meta-analysis of conjoint recognition experiments, which distinguished a semantic familiarity (gist trace based) factor, a context recollection (contextual trace based) factor, and a target recollection (verbatim trace based) factor. Based on this idea, there is a possibility that context trace retrieval along with target recollection failure are responsible for the false recognition of perceptually similar abstract shapes and that false recognitions are increased by adding a shared perceptual feature because it strengthens contextual trace. However, in the case of semantically related pictures, both context recollection and semantic familiarity may contribute to the false acceptance of related distractors as targets.

### Measurement models used in the conjoint recognition paradigm

As a measurement model and experimental procedure, a conjoint recognition (CR) paradigm was developed that disentangles verbatim and gist contributions to memory performance (Brainerd et al., 1999). In this paradigm, three types of test items (targets, related distractors, and unrelated distractors) are factorially combined with three types of recognition test instructions. In the initial version of the CR paradigm (Brainerd et al., 1999), the three instructional conditions expect: (1) accepting targets and rejecting both types of distractors, (2) accepting related distractors solely, and (3) accepting targets and related distractors and rejecting unrelated distractors. This experimental design requires three groups of participants, each group with a different recognition test instruction. However, more convenient within-subject versions of CR were later developed by Stahl and Klauer (2008, 2009) and Brainerd et al. (2010).

In CR paradigms, multinomial processing tree modelling is used as a measurement tool. This methodology can be applied to categorical data to measure hypothetical latent processes postulated by a particular theory (for reviews see Batchelder & Riefer, 1999; Erdfelder et al., 2009). The model can also serve to test the assumptions of a given theory. Multinomial models can be represented as decision trees connecting item types with overt responses through sequences of latent states, which are represented by branches in the tree structure. The great advantage of multinomial modelling is its capability of disentangling and estimating the separate contribution of underlying memory or decision processes to task performance (e.g. Bröder & Meiser, 2007; Nieznański, 2020).

The conjoint recognition paradigms differ in several assumptions and in the structure of the multinomial processing tree models, which is mainly due to the different response formats they use in the memory test. In the Stahl and Klauer’s (2009) model, which is referred to as the simplified CR model, participants are asked to classify each test item as either “target”, “related distractor”, or “unrelated distractor”. In the procedure designed by Brainerd et al. (2010) and its later modifications (Brainerd et al., 2022a), including a dual-recollection model (Brainerd et al., 2014), participants are required to respond “Yes” or “No” to three types of test probes: *Presented on the list?* (Target?), *Not presented on the list but related to presented material?* (Related?), and *Presented or not presented but related to presented material?* (Target or related?). For an individual participant, each test item is paired with one of these tree types of probes, and the assignment of probes is counterbalanced across participants. We will, hereinafter, refer to this model as the full CR model, to distinguish it from the simplified CR model. The differences between the multinomial models concern the number of parameters and processing trees structure. In comparison with the up-to-date Brainerd et al.'s (2022a) full CR model, Stahl and Klauer’s (2009) model does not include a parameter representing *erroneous recollection rejection* and requires imposing restrictions that eliminate one more parameter to make the model mathematically identifiable. Also, the different response formats lead to a different representation of response guessing. To verify that our results would hold independent of the analysis method, we used both the simplified (Experiments 1 and 2) and full CR models (Experiments 3–5). The differences between the models and their implications for the research conclusions will be discussed in more detail later in the article.

### Overview of the present experiments

**Fig. 1:**
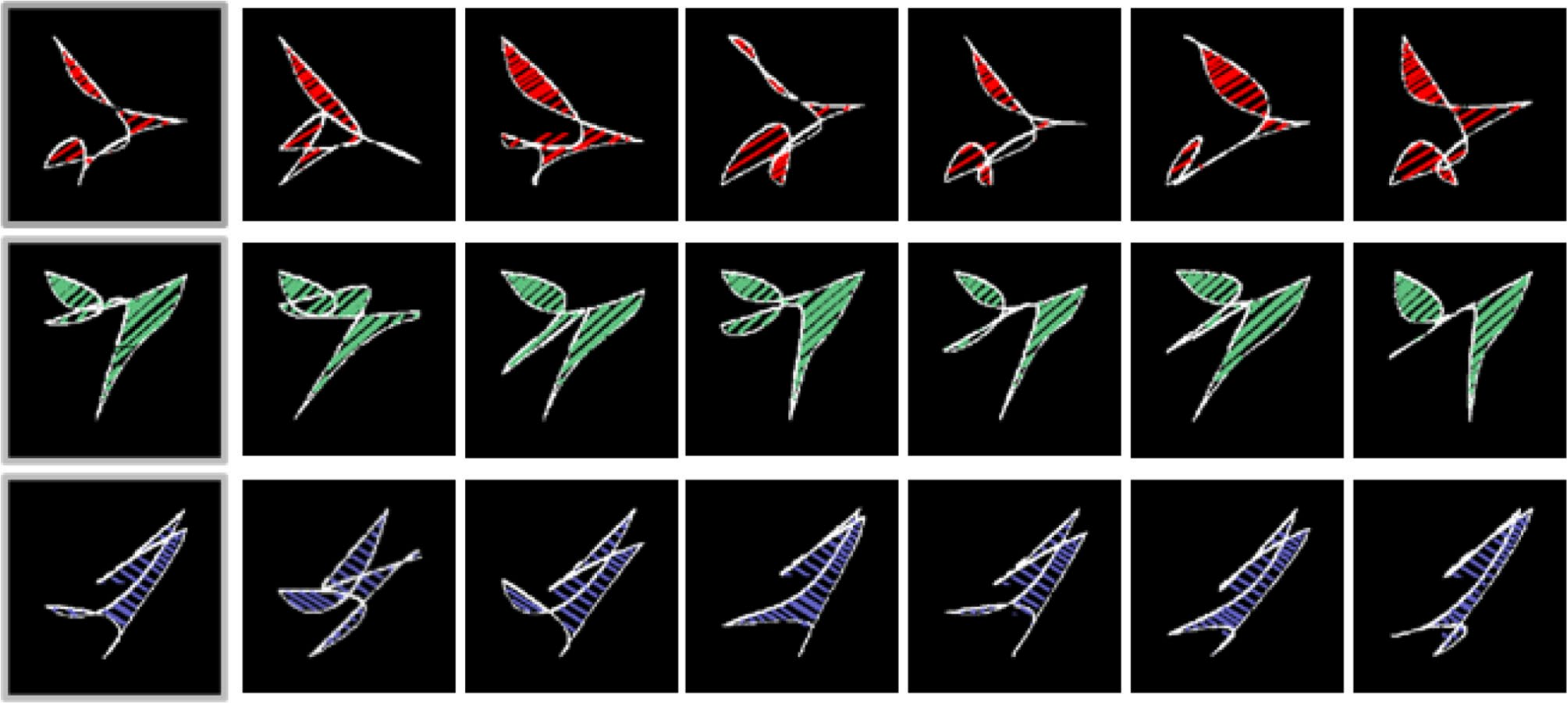
Examples of abstract shapes from Slotnick and Schacter (2004) used in Experiments 1, 3, and 5

**Fig. 2:**
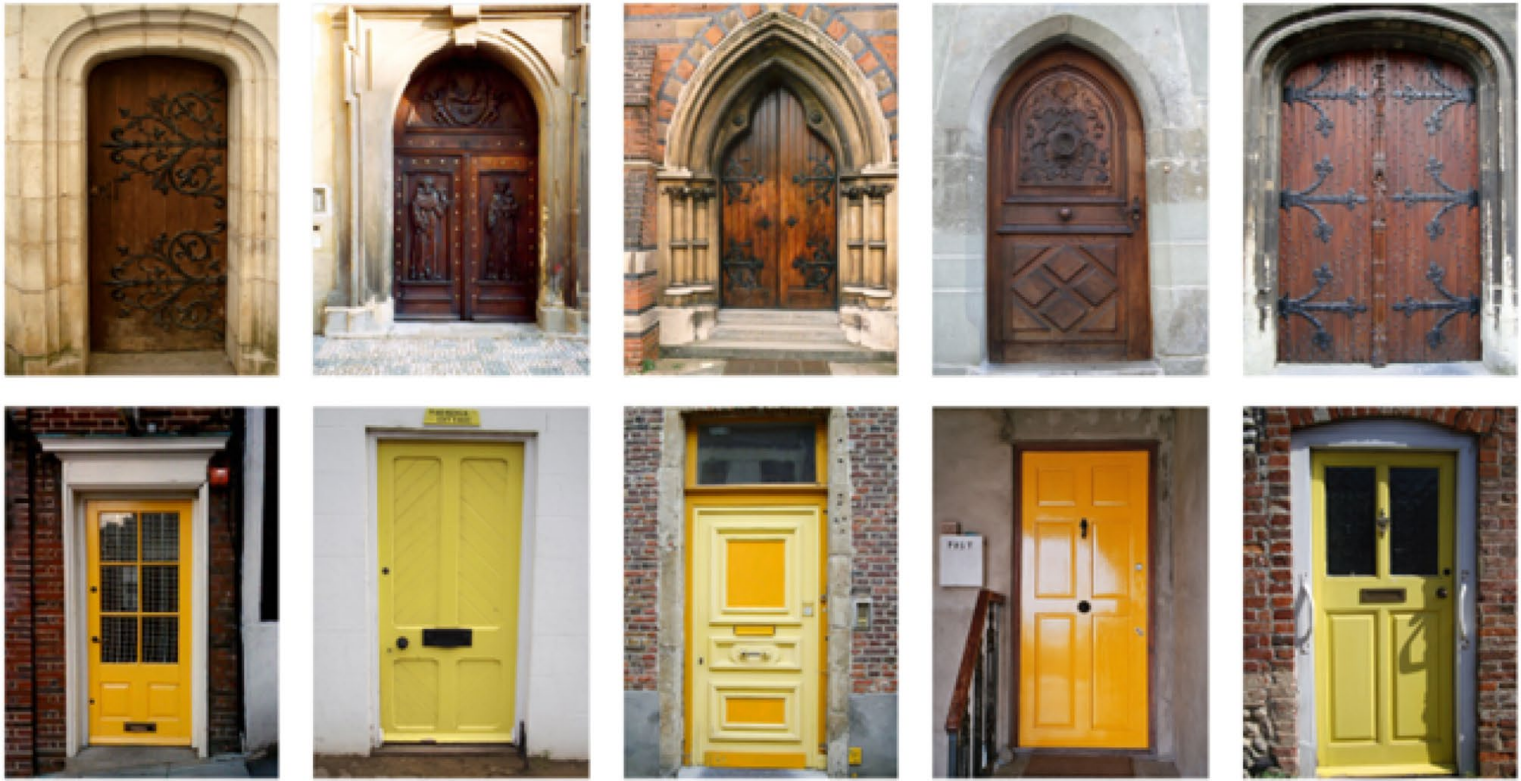
Examples of pictures of real objects from Baddeley et al. (2016) used in Experiments 2 and 4

In the five experiments we report, we investigated the processes underlying false memory for perceptually and/or conceptually similar distractors. We tested whether the processes defined in multinomial processing tree models of FTT as based on gist representation versus verbatim representation contribute to false acceptances of related distractors, depending on whether materials have or do not have pre-experimental semantic representations. Therefore, in Experiments 1, 3 and 5 we used as material abstract shapes (see Fig. 1 for examples), and for comparison, in Experiments 2 and 4, we used pictures of real objects (door scenes, see Fig. 2 for examples). In all experiments, we also introduced a manipulation of adding a surface feature (colour) to items belonging to the same class to test the consequences of increasing within-category perceptual homogeneity while decreasing between-category homogeneity. In one condition, all items in a category and their corresponding distractors, shared a common colour, in the other, all stimuli in all categories were presented in grey-scale. In addition, to test whether the results are robust to changes in the measurement model and procedure, we used two different paradigms. Experiments 1 and 2 involved the simplified CR model which defines parameters representing memory processes according to FTT, and uses a multiple-choice response format when testing memory. In contrast, in Experiments 3–5, we used the full CR model, which can be interpreted from both the perspective of FTT and the newer dual-recollection theory. In this case, the response format relies on accepting or rejecting the memory probe.

In sum, in Experiment 1, we studied the processes underlying false recognition of perceptually related abstract shapes using the simplified CR model, and then in Experiment 2, while leaving the model unchanged, we altered the material to pictures of real objects. In Experiment 3, we again used abstract shapes as materials, but changed the paradigm to the full CR model, and in Experiment 4, we used the full CR model and pictures of real objects. Finally, in a follow-up Experiment 5, we tested the possible role of subtle procedural changes for the inconsistent findings between Experiments 1 and 3.

## Experiment 1: False memory of abstract shapes in the simplified CR model

In the first experiment, we used abstract shapes (Fig. 1) that do not resemble animals or objects as materials to maximally reduce the contribution of semantic familiarity (i.e. gist trace retrieval) to memory performance. Shapes belonged to several categories of items created from a non-presented prototype (related distractor). We expected that adding a colour attribute to each category of shapes should result in higher false recognition of distractors related to this category due to phantom recollection (if it is not a gist-trace-but context-trace-based process). Alternatively, gist retrieval can contribute, if we redefine gist-trace as representing also perceptual patterns (or distinguish perceptual-gist from semantic-gist). Moreover, we can expect the impact of interference on the verbatim trace encoding to be greater in the grey-scale condition than in the colour condition, since targets sharing the grey-scale for all categories are generally more difficult to discriminate than targets differing in colours between categories.

In the colour condition, blocked presentation of study items belonging to the same category should increase phantom recollection (if surface features are represented in a context trace) or gist retrieval (if these features are encoded in a gist trace) and decrease verbatim trace encoding in comparison with the random order of presentation, where items from different categories are mixed. In studies using semantically related words it has been assumed that blocked presentation enhances gist processing, leading to an increase in false recall (e.g. Toglia et al., 1999) or the super-overdistribution phenomenon^1^ (Brainerd et al., 2019). However, experiments in the dual-recollection approach (Brainerd et al., 2015) indicated that the context recollection parameter was larger for blocked than for spaced presentation, with no effect on familiarity. In this experiment, as in our previous research with orthographically related words (Nieznański et al., 2019), we used the simplified CR model (Stahl & Klauer, 2008, 2009).

### Methods

#### Participants

Eighty first- and second-year psychology students were recruited from Cardinal Stefan Wyszyński University in Warsaw (12 males, the majority aged between 19 and 20 years old). The sample size was comparable in power to our earlier study (Nieznański et al., 2019), where a similar number of participants was sufficient to detect the effects of phonological similarity on memory parameters in the simplified CR model. Participants received extra credit points for their participation in the experiment. They were randomly assigned to the colour shapes/random order of presentation condition (*N* = 28), the grey-scale shapes/random order of presentation condition (*N* = 28) or the colour shapes/blocked order of presentation condition (*N* = 24). Data from two participants (one from each of the two random presentation conditions) who misunderstood the test instructions were discarded.

#### Stimuli

As materials we used lists of abstract shapes from Slotnick and Schacter (2004) study (available at *Attention, Memory, and Perception* (AMP) Lab, Boston College, web page https://sites.google.com/bc.edu/sd-slotnick/publications/scripts-and-stimuli). The original set of stimuli consisted of prototype shapes and series of distinct exemplars of these prototypes that were spatially distorted versions of prototype shapes. The procedure of stimuli selection used by Slotnick and Schacter (2004) rejected shapes that were similar to animals or objects. The shapes within each prototype-exemplar set were filled-in using the same specific colour and line orientation. In Experiment 1, we selected 24 prototype shapes with eight exemplars for each prototype (192 exemplars in total). The same stimuli were used in the colour and grey-scale conditions with the exception that all stimuli in the grey-scale condition were filled-in using grey-scale instead of original colours. The set of stimuli was split into four subsets for counterbalancing purposes. At test, targets or prototypes were taken from two subsets of stimuli and unrelated distractors from the other two subsets. When a prototype related to a given list was presented at test, an exemplar from this list was not presented, and vice versa when an exemplar was presented, the prototype was omitted. This eliminated instances in which consecutive presentation of a target and distractor from the same list at test would influence participants’ recognition decisions (cf. Brainerd et al. 1995b).

#### Design and procedure

The participants were examined at individual workstations in the University Lab. The presentation of the stimuli and the response recording were conducted using the E-Prime programme 2.0 (Psychology Software Tools, Pittsburgh, PA).

At the beginning, the participants were instructed to remember abstract shapes. The whole study list consisted of 96 colour or grey-scale shapes (12 sets of 8 exemplars each), presented one by one in random order or blocked for 3000 ms each, followed by a blank slide presented for 250 ms. Before the test phase, participants solved simple addition problems as a short filler task. Test lists consisted of 24 shapes: 6 targets, 6 prototypes, and 12 non-presented distractors (i.e. 6 targets and 6 prototypes from the non-presented lists). Following the simplified CR paradigm, participants were instructed to recognize whether the shape was presented on the study list, was not presented but is very similar to one of the presented shapes, or is entirely new. At test, slides were presented in random order at self-paced rate. The study condition (colour/random, colour/blocked, and grey-scale/random) was manipulated between-subjects, the type of test stimuli (target, prototype, distractor) was manipulated within-subjects.

#### Multinomial model for the simplified CR paradigm

In Experiments 1 and 2, we used the model tailored for the simplified CR paradigm by Stahl and Klauer (2009). Figure 3 depicts the multinomial processing trees for three item types: targets, related distractors, and unrelated distractors. Participants’ responses, placed on the right side, are connected with item types by branches of the trees that represent latent processes defined according to the FTT. The processes represent the probability of verbatim trace retrieval when a target is presented at test (*V*_t_), as well as the probability of retrieving the verbatim trace when a related distractor is presented at test (*V*_r_). The latter process, called *recollection rejection* leads to “related” response and occurs when a related distractor cues retrieval of the corresponding target. A “target” response to a related distractor may occur in the absence of recollection rejection due to *phantom recollection* (*P*_r_), which is a vivid but false recollective experience of distractor’s presentation at study. Then, the probability of retrieving the gist trace is represented by parameters *G*_t_ or *G*_r_, when a target or related distractor is presented at test, respectively. Given that no verbatim or gist memory is available for the test item, the observer may guess that it is old with a probability of *b*. Finally, parameter *a* is the probability of guessing that this undetected item is a target. The full version of Stahl and Klauer’s (2009) model contains seven parameters (*V*_t_,* V*_r_,* P*_r_, *G*_t_, *G*_r_, *a*, *b*) which is too many in relation to six degrees of freedom in the data for one experimental condition. To overcome this problem of model non-identifiability, restrictions have to be imposed on the parameters. Following Stahl and Klauer’s (2009) recommendation for DRM-like paradigms, we chose an assumption that the recollection rejection parameter can be eliminated in the model. It is plausible that it is difficult for a participant to exhaustively retrieve verbatim traces of all the presented list items and reject the related distractor because it is not included in this list (cf. Gallo, 2004; Gong et al., 2016; Nieznański et al., 2019).

**Fig. 3:**
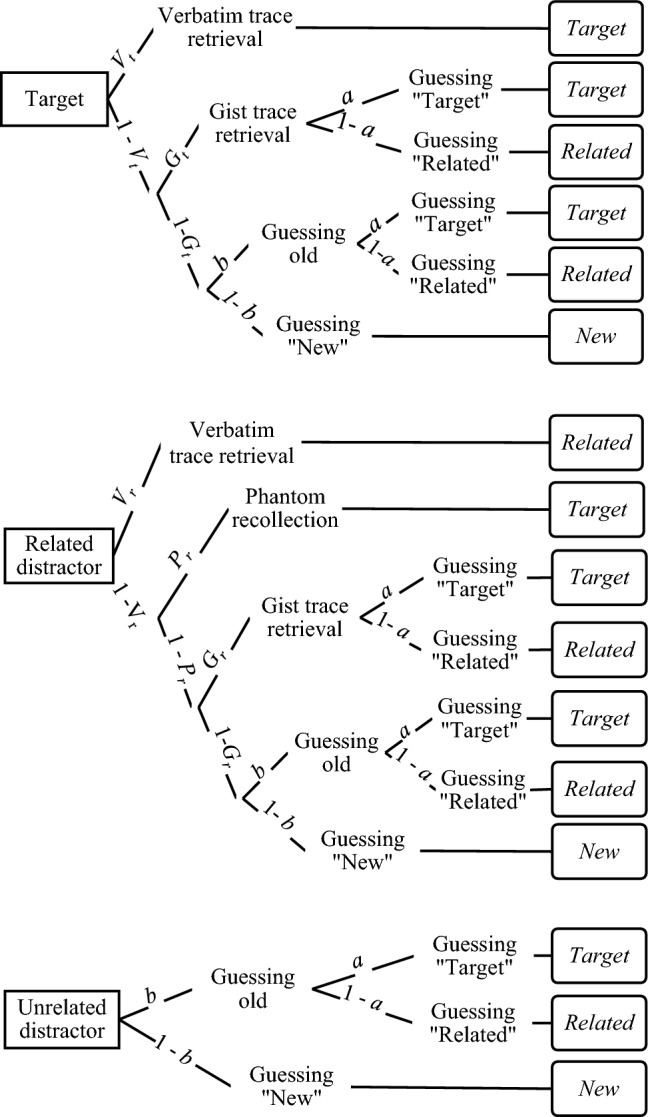
Multinomial processing tree for the simplified CR model by Stahl and Klauer (2009)

The goodness of fit of the model to the empirical data was tested with the log-likelihood ratio statistic (*G*^2^), which is distributed asymptotically as a *χ*^2^ distribution. At α level of 0.05, *G*^2^(1) = 3.84 indicates a critical value. Computations were carried out with the *multiTree* computer programme (Moshagen, 2010). A post hoc sensitivity power analysis conducted using G*Power 3 (Faul et al., 2007) indicated that the number of 1872 observations gathered in this experiment, allowed to detect a small effect size *w* = 0.07, with satisfactory test power of 1 − β = 0.80 for parameter comparisons across the three conditions (*df* = 2). Pooled over participants, the response frequencies obtained in this and other experiments and used for multinomial modelling analyses are reported in “Appendix” section.

## Results and discussion

### Results based on descriptive measures

**Fig. 4:**
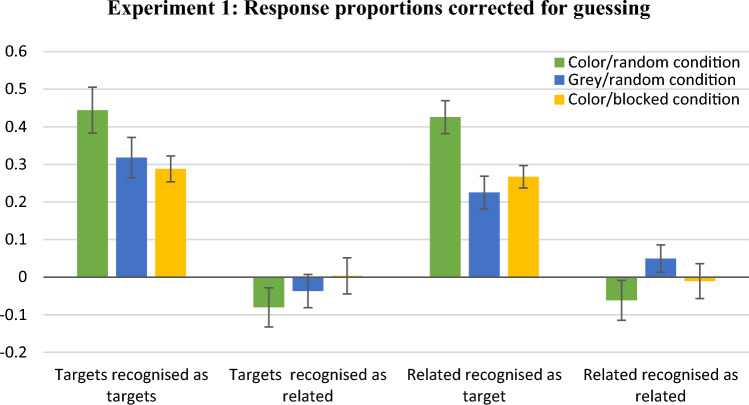
Mean proportions of response types in Experiment 1 with abstract shapes as materials. Bars show the proportions of particular responses to targets or related distractors, minus the corresponding proportions of responses to unrelated distractors. Error bars represent standard errors

Figure 4 presents the mean proportions of response types corrected for guessing (e.g. the proportion of “target” response to targets minus the proportion of “target” response to unrelated distractors). We planned comparisons in pairs of the colour/random condition with the grey-scale/random condition and with the colour/blocked condition (the grey-scale/random condition and the colour/blocked condition were not compared with each other since they differ simultaneously on two factors). The results showed that non-presented prototypes were significantly more often accepted as targets (after correction for guessing) in the colour/random (*M* = 0.43, SD = 0.23) than in the grey-scale/random condition (*M* = 0.22, SD = 0.23), *t*(52) = 3.24, Cohen’s *d* = 0.88, *p* = 0.002. The proportion of accepting prototypes as targets was also higher in the colour/random than in the colour/blocked condition (*M* = 0.27, SD = 0.14), *t*(44.65) = 2.99, Cohen’s *d* = 0.82, *p* = 0.004. In the colour/random condition proportion of recognitions of targets as targets was significantly higher (*M* = 0.44, SD = 0.32) than in the colour/blocked condition (*M* = 0.29, SD = 0.17), *t*(40.52) = 2.23, Cohen’s *d* = 0.60, *p* = 0.03. Comparisons of the proportions of targets recognised as related and prototypes recognised as related did not differ between conditions, however, these proportions were very low, suggesting possibility of a floor effect. In sum, descriptive measures indicate that accurate identifications of targets were best in the colour/random condition and, at the same time, in this condition false recognitions of related distractors as targets was highest.

### Results based on multinomial modelling

For the purposes of Experiment 1, we constructed a combined multinomial model for the three conditions: the colour/random condition, the grey-scale/random condition, and the colour/blocked condition. As mentioned, to make the model mathematically identifiable, we assumed that the recollection rejection parameter *V*_r_ is equal to zero in all conditions. The parameter estimates are presented in the upper half of Table 1. The far right column of the table shows the change in model fit between the baseline model and the model with a given parameter equalized across the three conditions.

**Table 1:** Parameter estimates and standard errors of the simplified CR model in Experiments 1 and 2

Parameter	Colour/random	Grey/random	Colour/blocked	Comparison
Experiment 1: Abstract shapes				
Verbatim trace retrieval (*V*_t_)	**0.49 (0.06)**	0.35 (0.07)	**0.29 (0.07)**	Δ*G*^2^(2) = 5.46, *p* = 0.06
Gist trace retrieval for targets (*G*_t_)	0.41 (0.11)	0.37 (0.11)	0.38 (0.11)	Δ*G*^2^(2) = 0.08, *ns*
Phantom recollection (*P*_r_)	**0.46 (0.06)**	**0.18 (0.08)**	**0.27 (0.07)**	Δ*G*^2^ (2) = 8.83, *p* = 0.01
Gist trace retrieval for related (*G*_r_)	0.44 (0.11)	0.48 (0.10)	0.30 (0.11)	Δ*G*^2^(2) = 1.56, *ns*
Guessing “target” (*a*)	0.38 (0.04)	0.46 (0.04)	0.43 (0.04)	Δ*G*^2^(2) = 1.69, *ns*
Guessing old (*b*)	0.48 (0.03)	0.52 (0.03)	0.49 (0.03)	Δ*G*^2^(2) = 1.44, *ns*
Experiment 2: Door scenes				
Verbatim trace retrieval (*V*_t_)	0.77 (0.02)	0.70 (0.03)		Δ*G*^2^(1) = 3.40, *ns*
Gist trace retrieval for targets (*G*_t_)	0.81 (0.05)	0.73 (0.05)		Δ*G*^2^(1) = 1.47, *ns*
Phantom recollection (*P*_r_)	**0.23 (0.04)**	**0.11 (0.04)**		Δ*G*^2^(1) = 4.50, *p* = 0.03
Gist trace retrieval for related (*G*_r_)	**0.69 (0.03)**	**0.54 (0. 04)**		Δ*G*^2^(1) = 8.61, *p* = 0.003
Guessing “target” (*a*)	0.18 (0.04)	0.26 (0.03)		Δ*G*^2^(1) = 2.83, *ns*
Guessing old (*b*)	**0.18 (0.01)**	**0.30 (0.02)**		Δ*G*^2^(1) = 27.37, *p* < 0.001

Significantly different results are marked in bold

We started our analysis by testing the FTT assumption that gist traces do not represent non-semantic features of abstract shapes. However, we found that the parameters of gist trace retrieval for related distractors were well above zero (the null hypothesis that *G*_r_ = 0 was rejected in all conditions, Δ*G*^2^(1)s > 5.70, *p*s < 0.02), and the same was true for contribution of gist retrieval to target recognition, Δ*G*^2^(1)s > 7.61, *p*s < 0.006. These results are consistent with our previous observation that gist retrieval cannot be ruled out as a process contributing to the false recognition of orthographically related words (Nieznański et al., 2019).

Concerning the role of adding a shared surface feature, we found that the verbatim trace retrieval parameter was significantly higher in the colour/random than blocked condition, Δ*G*^2^(1) = 5.17, *p* = 0.02, which suggests that the blocked presentation of similar shapes caused interference between verbatim representations. Moreover, phantom recollection was significantly higher in the colour/random condition than in the blocked condition, Δ*G*^2^(1) = 4.23, *p* = 0.04, and in the grey-scale condition, Δ*G*^2^(1) = 8.46, *p* = 0.004. The gist trace retrieval parameter for non-presented prototypes was not different between conditions, therefore, we can conclude that the higher level of false recognitions of similar shapes in the colour/random condition was due to increased phantom recollection. This result is consistent with Brainerd et al.'s (2022a) approach suggesting that contextual details of multiple items are represented in a context trace. The contribution of phantom recollection to false recognition of related distractors can be interpreted as resulting from stronger context trace in the colour/random condition.

The prediction of an increase in the contribution of gist retrieval or phantom recollection in the blocked as compared to random condition was not confirmed in our experiment. Results based on descriptive measures also indicated that blocked presentation does not increase false recognition. In general, manipulations involving blocking and adding colour to categories increase within-category homogeneity and decrease between-category homogeneity in comparison with random and grey-scale presentations. An increase in *within*-category homogeneity should lead to an increase in false alarms to *related* distractors that have category-specific features. This prediction was confirmed for the colour manipulation, but not for the blocked presentation manipulation, as we found no increase in false alarms to related distractors in the blocked condition. Enhanced distinctiveness *between* the categories should lead to a decrease in false alarms to *unrelated* distractors which do not have category-specific features, and can be rejected on this basis (Nieznański & Obidziński, 2022). From the perspective of the context noise model of recognition memory, one can predict that it is easier for participants to form separate category representations and incorporate them into the contextual representation under blocked rather than random presentation conditions. More specific category representations make participants more capable of excluding distractors unrelated to these categories (Dennis & Chapman, 2010). However, our results do not support this prediction, as we found no significant differences in the proportions of false alarms to unrelated distractors between experimental conditions. This may be due to the specificity of stimuli used in this experiment, which are novel to the participants, so forming non-overlapping representations at study and then reinstating them at test may be ineffective even with blocked presentation (cf. Kinnell & Dennis, 2012).

Our observation of higher phantom recollection in the *random* condition than in the blocked condition for abstract shapes is at odds with the effects reported in the literature for verbal material (Brainerd et al., 2015). This dissociation between effects observed for semantically versus visually related materials extends the conclusion of Chang and Brainerd (2021) that semantic and phonological similarities are processed in different ways. While the blocked presentation of semantically related words makes it easier to find similarities, the blocked presentation of items that share the same perceptual feature may provoke their separation and perhaps lead to a search for differentiating features.

## Experiment 2: False memory of door scenes in the simplified CR model

In the first experiment, we found that adding a surface feature that is shared by stimuli belonging to the same category leads to increase in false memory for non-presented related distractors. The basis for category membership was the similarity of abstract shapes, therefore, they possessed common perceptual features but did not have a common meaning. However, it is possible that providing a new perceptual feature can enhance common gist extraction when the stimuli belonging to a particular category are meaningful pictures or—in other words—this common perceptual feature can be encoded into gist trace, enhancing its contribution to performance (cf. Chen et al., 2018). In Experiment 2, we tested the effects of presenting door scenes (Fig. 2) organized into semantic categories that share colours within these categories on false memory for related distractors. In this and the subsequent experiments, we no longer intended to examine the effect of blocking and therefore all stimuli were presented in random order.

### Methods

#### Participants

Seventy second-year psychology students (12 males, median age 20 years) were recruited from Cardinal Stefan Wyszyński University in Warsaw. They received extra credit points for their participation in the experiment. Participants were randomly assigned to the colour condition (*N* = 35) or the grey-scale condition (*N* = 35).

#### Stimuli

We used 80 full-colour photographs of door scenes as materials. Most of these were selected from a computerized database prepared by Baddeley et al. (2016), in which the door scenes are categorized along a set of various dimensions. We selected the stimuli on the basis of the features from two dimensions: function and colour. The stimuli belonged to the following categories—10 domestic ordinary: yellow, 10 garage: red, 10 church: brown, 10 business premise: green, 10 beach hut: blue, and 10 post-office: white or glass, and 20 from other categories, multicolour or in different colours than those in the target categories. Examples of pictures from the categories: church–brown and domestic–yellow are shown in Fig. 2. For the second experimental condition, grey-scale versions of each picture were generated. All pictures were in 600/789 pixels format. In comparison with Experiment 1, we found that in Experiment 2 creating distinct stimulus categories was more challenging, therefore, the stimulus list was shorter, and unrelated distractors were not selected from separate lists of items.

#### Design and procedure

First, participants were instructed to remember door scenes. The study list consisted of 50 pictures (6 sets of 8 exemplars each plus two buffers at the beginning) presented in random order for 3000 ms each, followed by a blank slide presented for 250 ms. Before the test phase, participants solved simple addition problems as a filler task. In comparison with Experiment 1, not one but two targets and two related distractors were taken from each category, and both targets and related distractors from each list were presented. This strategy of item selection was intended to increase the number of stimuli with a limited number of available categories. The test lists consisted of 42 pictures: 12 targets (2 exemplars from 6 categories), 12 related distractors (2 non-presented exemplars from six categories), and 18 unrelated distractors. For counterbalancing purposes, the two pictures that were selected to be the targets presented at test were switched with the two corresponding related distractors for half of the participants. At test, participants were instructed to recognize whether the picture was presented on the study list, was not presented but is very similar to one of the presented door scenes, or is entirely new. The slides were presented in a random order, at a self-paced rate.

The colour versus grey-scale condition was manipulated between-subjects, and the type of test stimuli (target, related distractor, unrelated distractor) was manipulated within-subjects. The experiment was carried out in a university lab using the E-Prime 2.0 programme. A post-hoc sensitivity power analysis (conducted using G*Power 3, Faul et al., 2007) showed that the test for difference between parameters in the colour and grey-scale conditions had a satisfactory power of 1 − β = 0.80 to detect a small effect size *w* = 0.05, given an alpha level of 0.05, *df* = 1, and the total number of 2940 observations gathered in this experiment.

## Results and discussion

### Results based on descriptive measures

**Fig. 5:**
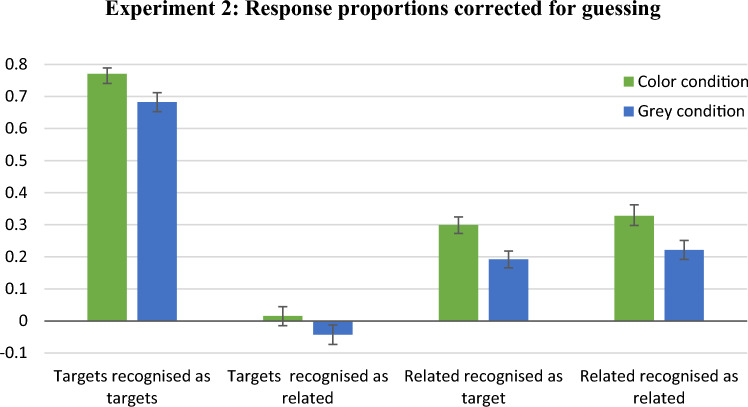
Mean proportions of response types in Experiment 2 with door scenes as materials. Bars show the proportions of particular responses to targets or related distractors, minus the corresponding proportions of responses to unrelated distractors. Error bars represent standard errors

Figure 5 presents the mean proportions of response types corrected for guessing. The corrected-proportion of “target” responses to targets in the colour condition (*M* = 0.77, SD = 0.11) was higher than in the grey-scale condition (*M* = 0.68, SD = 0.18), Mann–Whitney *U* = 429.00, *z* = 2.16, *p* = 0.03. Further, the corrected-proportion of “target” responses to related distractors was higher in the colour condition (*M* = 0.30, SD = 0.15) than in the grey-scale condition (*M* = 0.19, SD = 0.16), *t*(68) = 2.95, Cohen’s *d* = 0.70, *p* = 0.004. The corrected proportion of “similar” responses to related distractors was higher in the colour condition (*M* = 0.33, SD = 0.20) than in the grey-scale condition (*M* = 0.22, SD = 0.18), *t*(68) = 2.34, Cohen’s *d* = 0.56, *p* = 0.02. Consistent with Experiment 1, this experiment indicated better identification of targets for colourful than grey-scale stimuli at cost of more frequent false recognition of related distractors as targets. In contrast with Experiment 1, the correct recognition of related distractors as similar to presented stimuli was better in the colour than grey-scale condition.

### Results based on multinomial modelling

As in Experiment 1, a multinomial model based on the simplified CR paradigm (Stahl & Klauer, 2009) was created. As done previously, to make the model identifiable, we assumed that the recollection rejection parameter *V*_r_ is equal to zero. The parameter estimates are presented in the bottom half of Table 1. We found that the phantom recollection parameter was significantly higher in the colour condition than in the grey-scale condition. In a similar vein, the gist trace retrieval parameter for related distractors was significantly higher in the colour condition than in the grey-scale condition. Moreover, participants were more prone to guess that the test item is not new in the grey-scale condition than in the colour condition.

In sum, using concrete pictorial materials, this experiment showed that related distractors are more often falsely recognised as targets when category members share the same colour in comparison with the condition when all items are presented in grey-scale. Multinomial modelling analyses indicated that this increase in false recognitions is both due to phantom recollection and gist retrieval processes. In Experiment 1, where abstract shapes were used, only phantom recollection contributed to the increase in false recognitions of related distractors. This supports the assumption that the additional feature added to multiple items is represented in the context trace (Brainerd et al., 2022a) and that the surface details associated with a category are stored along with the semantic information (Chen et al., 2018).

## Experiment 3: False memory of abstract shapes in the full CR model

In Experiments 1 and 2, we used the simplified CR model which required imposing restrictions on the recollection rejection parameter. Eliminating this parameter did not lead to model rejection, suggesting that its contribution to memory performance was negligible. However, FTT and dual-recollection theory postulate that interference with the verbatim suppression process, or recollection rejection, is the main source of false acceptances of non-semantically related distractors (Chang & Brainerd, 2021). Therefore, it is desirable to study false memory using a model that does not impose constraints on the recollection rejection parameter. Therefore, in our third experiment, we applied a full CR model (Brainerd et al., 2022a) since it does not require any restrictions on the model parameters. Following the FTT and the dual-recollection theory, it can be expected that the semantic familiarity parameters contribution to the recognition of abstract shapes should be negligible, and that adding a surface feature to items belonging to one category should increase processes based on context trace retrieval (i.e. phantom recollection or erroneous recollection rejection).

### Methods

#### Participants

Seventy-three second-year psychology students (13 males, median age 20 years) were recruited from Cardinal Stefan Wyszyński University in Warsaw. They received extra credit points for their participation in the experiment. Participants were randomly assigned to the colour condition (*N* = 37) or the grey-scale condition (*N* = 36).

#### Stimuli

Similar to Experiment 1, we used abstract shapes from Slotnick and Schacter’s (2004) study as materials. Items were selected from 18 lists divided into two sets of 9 lists. From the first set of lists, 10 items were selected from each list. Six of them (from positions 3–8 in the original database) were assigned to be targets presented at study. The prototype and two other shapes (from positions 0, 1, and 2, respectively) were assigned to be related distractors presented only at test. One item from each of 9 lists (position 9) was used as a buffer—5 were presented at the beginning, and 4 at the end of the study list. As unrelated distractors items from the second set of lists were used—these were prototypes and shapes from positions 1 and 2 of these nine lists.

#### Design and procedure

Participants were instructed to try to remember abstract shapes belonging to some categories. The study list consisted of 63 shapes (9 lists × [6 targets + 1 buffer]), presented in random order for 3000 ms each, followed by a blank slide presented for 300 ms.

Before the test phase, participants were informed that their task was to distinguish the same items from new-similar and new-unrelated items. They were given several examples of shapes to explain what is meant by similar versus unrelated items. The test list consisted of 81 shapes: 27 targets (9 presented lists × 3 exemplars, taken from positions 3, 4, and 5), 27 related distractors (9 presented lists × [1 prototype + 2 list items]), 27 unrelated distractors (9 unpresented lists × [1 prototype + 2 list items]).

In line with the full CR paradigm, participants were informed that their task was to answer “yes” or “no” to the question presented under the test picture on a particular slide. There were three types of probe questions counterbalanced across test items: (a) Target? probes (T?): *Was this shape presented?*; (b) Related? probes (R?): *Was this shape not presented but is similar to the presented shapes?*; and (c) Target or Related? probes (TorR?): *Was this shape presented or is similar to the presented shapes?* At test, slides were presented in random order and at a self-paced rate. The colour condition versus the grey-scale condition was manipulated between-subjects. The experiment was carried out at a university lab using E-Prime 2.0.

A post-hoc sensitivity power analysis (conducted using G*Power 3, Faul, et al., 2007) showed that the test for the difference between model parameters in the colour and grey-scale conditions had a satisfactory power of 1 − β = 0.80 to detect a small effect size *w* = 0.04, given an alpha level of 0.05, *df* = 1, and the total number of 5913 observations gathered in this experiment.

#### Multinomial model for the full CR paradigm

In Experiments 3, 4, and 5, we used the multinomial model designed for the full CR paradigm by Brainerd et al., (2022a). This model is depicted in Fig. 6 and contains three multinomial processing trees for targets and three for related distractors (another three trees for unrelated distractors are not presented). For target cues, retrieving verbatim traces produces correct acceptance on T? or TorR? probes or correct rejection on R? probes with probability represented by the *identity* parameter. When identity fails, targets can generate a kind of ersatz recollection, represented by the *erroneous recollection rejection* parameter, that results in incorrect rejection on T? probes and incorrect acceptance on R? probes, but correct acceptance on ToR? probes. Originally, this parameter was interpreted as instances when targets cue retrieval of *verbatim traces* of other targets (Brainerd et al., 2003), however, the dual-recollection interpretation supported by a recent meta-analysis (Brainerd et al., 2022a) indicated that this parameter depends on context trace retrieval. Finally, when identity and erroneous recollection rejection both fail, targets may be accepted on the basis of gist trace retrieval that is represented by the *semantic familiarity* parameter.

**Fig. 6:**
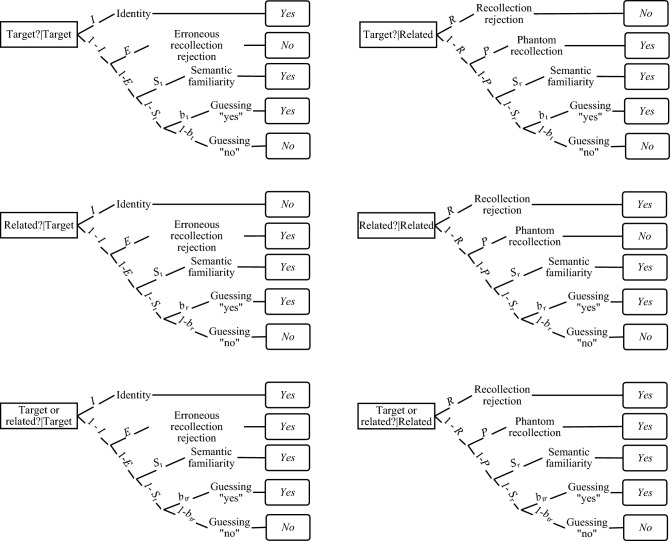
Multinomial processing trees for the full CR model by Brainerd et al., (2022a)

In the case of related distractors, the model assumes that they are correctly rejected on T? probes or correctly accepted on R? and TorR? probes on the basis of verbatim trace retrieval represented by the *recollection rejection* parameter. When recollection rejection fails, the *phantom recollection* parameter leads to false acceptance of related distractors on T? probes, incorrect rejections on R? probes, and correct acceptances on TorR? probes. Initially, this illusory recollection was interpreted as based on strong gist retrieval (Brainerd et al., 2001), however, the dual recollection model treats this parameter as measuring the retrieval of traces of contextual details (Brainerd et al., 2022a). When recollection rejection and phantom recollection both fail, *gist trace* retrieval results in the acceptance of related distractors, which is represented by the *semantic familiarity* parameter. For both targets and related distractors, when all memory processes fail, probes may be accepted on the basis of guessing, and the *response bias* parameters are specific for the probe type. In the full CR model, the acceptance of unrelated distractors is based solely on the response bias.

## Results and discussion

### Results based on descriptive measures

**Fig. 7:**
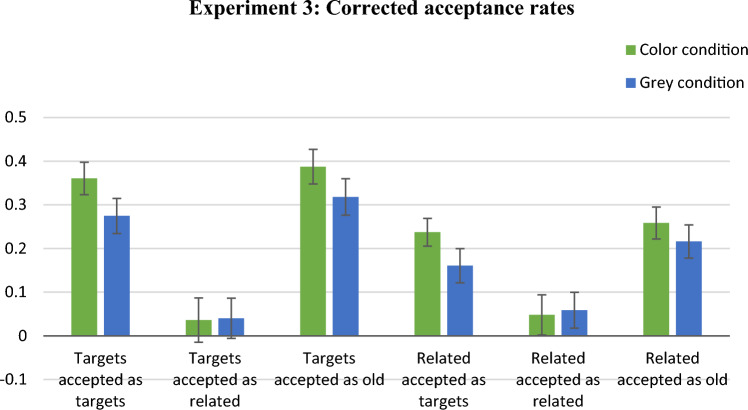
Mean proportions of response types in Experiment 3, using abstract shapes. Bars show the proportions of acceptances of test probes for targets or related distractors, minus the proportions of acceptances of the same probes for unrelated distractors. Error bars represent standard errors

Figure 7 presents the corrected-for-guessing mean acceptance proportions for all probe-response types. Although the patterns are similar to the results of Experiment 1, none of the differences between conditions reached statistical significance. We came to the same conclusions after computing the results separately for prototypes and list items used as related distractors—no significant difference was found.

We also compared all corrected acceptance rates between conditions using Bayesian Mann–Whitney *U* tests. These analyses were conducted in JASP (JASP Team, 2019), and yielded weak or moderate evidence for the null hypotheses (0.235 < BF_10_ < 0.670). In particular, when comparing the false acceptances of related distractors as targets, the Bayes factor (BF_10_ = 0.670) indicated weak evidence for the hypothesis that the acceptance proportions for the colour and grey-scale condition are equal.

### Results based on multinomial modelling

The parameter estimates of the full CR multinomial model are presented in the upper part of Table 2. First, we tested whether the parameters representing semantic familiarity for targets (*S*_t_) or related distractors (*S*_r_) are equivalent to zero, we rejected this hypothesis for all conditions, Δ*G*^2^(1)s > 8.45, *p*s < 0.004. As for the recollection rejection parameter (*R*), although numerically it turned out to have low contribution to performance (< 0.13), its values were significantly greater than zero, for both the colour and grey-scale conditions, Δ*G*^2^(1)s > 4.19, *p*s < 0.04. Second, we found no significant difference for the model parameters between the colour and grey-scale conditions. In particular, no difference was found for the phantom recollection parameter, which differed significantly between conditions in Experiment 1, using the same abstract materials. Note, since more observations were gathered for the colour and the grey-scale conditions in this experiment (5913) than in Experiment 1 (1296), the power to detect an effect was even higher in Experiment 3.

**Table 2:** Parameter estimates and standard errors of the full CR model in Experiments 3, 4 and 5

Parameter	Colour condition	Grey condition	
Experiment 3: Abstract shapes			
Identity (*I*)	0.30 (0.03)	0.24 (0.04)	Δ*G*^2^(1) = 1.20, *ns*
Erroneous recollection rejection (*E*)	0.22 (0.06)	0.19 (0.06)	Δ*G*^2^(1) = 0.10, *ns*
Semantic familiarity for targets (*S*_t_)	0.39 (0.05)	0.31 (0.05)	Δ*G*^2^(1) = 1.59, *ns*
Recollection rejection (*R*)	0.11 (0.05)	0.13 (0.05)	Δ*G*^2^(1) = 0.07, *ns*
Phantom recollection (*P*)	0.19 (0.05)	0.15 (0.05)	Δ*G*^2^(1) = 0.35, *ns*
Semantic familiarity for new-similar (*S*_r_)	0.23 (0.05)	0.18 (0.05)	Δ*G*^2^(1) = 0.53, *ns*
Guessing “yes” for Target? probes (*b*_t_)	0.23 (0.02)	0.27 (0.02)	Δ*G*^2^(1) = 1.43, *ns*
Guessing “yes” for Similar? probes (*b*_r_)	0.50 (0.03)	0.51 (0.03)	Δ*G*^2^(1) = 0.08, *ns*
Guessing “yes” for Target or Similar? probes (*b*_tr_)	0.42 (0.03)	0.45 (0.03)	Δ*G*^2^(1) = 0.49, *ns*
Experiment 4: Door scenes			
Identity (*I*)	0.38 (0.03)	0.44 (0.03)	Δ*G*^2^(1) = 2.21, *ns*
Erroneous recollection rejection (*E*)	0.24 (0.06)	0.21 (0.07)	Δ*G*^2^(1) = 0.08, *ns*
Semantic familiarity for targets (*S*_t_)	0.51 (0.04)	0.43 (0.05)	Δ*G*^2^(1) = 1.89, *ns*
Recollection rejection (*R*)	0.29 (0.05)	0.24 (0.05)	Δ*G*^2^(1) = 0.55, *ns*
Phantom recollection (*P*)	0.17 (0.06)	0.14 (0.07)	Δ*G*^2^(1) = 0.10, *ns*
Semantic familiarity for new-similar (*S*_r_)	**0.44 (0.05)**	**0.24 (0.06)**	**Δ*G***^**2**^**(1) = 6.90, *p* < 0.01**
Guessing “yes” for Target? probes (*b*_t_)	0.08 (0.02)	0.10 (0.02)	Δ*G*^2^(1) = 0.46, *ns*
Guessing “yes” for Similar? probes (*b*_r_)	0.40 (0.03)	0.38 (0.03)	Δ*G*^2^(1) = 0.25, *ns*
Guessing “yes” for Target or Similar? probes (*b*_tr_)	0.20 (0.03)	0.26 (0.03)	Δ*G*^2^(1) = 2.27, *ns*
Experiment 5: Abstract shapes			
Identity (*I*)	0.16 (0.08)	0.37 (0.08)	Δ*G*^2^(1) = 3.32, *ns*
Erroneous recollection rejection (*E*)	0.07 (0.12)	0.15 (0.16)	Δ*G*^2^(1) = 0.16, *ns*
Semantic familiarity for targets (*S*_t_)	0.36 (0.08)	0.21 (0.13)	Δ*G*^2^(1) = 1.17, *ns*
Recollection rejection (*R*)	0.00 (0.10)	0.04 (0.10)	Δ*G*^2^(1) = 0.17, *ns*
Phantom recollection (*P*)	0.25 (0.09)	0.25 (0.10)	Δ*G*^2^(1) = 0.00, *ns*
Semantic familiarity for new-similar (*S*_r_)	0.28 (0.09)	0.32 (0.09)	Δ*G*^2^(1) = 0.14, *ns*
Guessing “yes” for Target? probes (*b*_t_)	0.29 (0.04)	0.31 (0.04)	Δ*G*^2^(1) = 0.22, *ns*
Guessing “yes” for Similar? probes (*b*_r_)	0.53 (0.04)	0.52 (0.04)	Δ*G*^2^(1) = 0.06, *ns*
Guessing “yes” for Target or Similar? probes (*b*_tr_)	0.50 (0.04)	0.54 (0.04)	Δ*G*^2^(1) = 0.59, *ns*

Significant results are marked in bold

In sum, Experiment 3 did not support the observation from Experiment 1, that adding a shared colour to stimuli from an abstract category leads to differences in accurate or false recognition in comparison with the grey-scale condition. It seems that the change in the paradigm from the simplified CR to the full CR may have led to this difference. However, it should be noted, that apart from the paradigm differences, there were also some subtle differences in procedure and in the selection of related distractors in the testing phase that may have played a role. Before addressing this issue in Experiment 5, we will present the results of an experiment conducted in full CR with concrete materials.

## Experiment 4: False memory of door scenes in the full CR model

In the fourth experiment, we expected to replicate the results of Experiment 2, that is, an increase in phantom recollection and semantic familiarity in the colour condition, but using the full CR model instead of the simplified CR model.

### Methods

#### Participants

Fifty second-year psychology students were recruited from Cardinal Stefan Wyszyński University in Warsaw (4 males, median age 21 years). They received extra credit points for their participation in the experiment. Participants were randomly assigned to the colour condition (*N* = 25) or the grey-scale condition (*N* = 25). Data from four participants (two from each condition) who misunderstood the test instructions or were identified as outliers based on their responses to unrelated distractors (their acceptance rate was about 3 times higher than the group mean) was excluded.

#### Stimuli

In this experiment, we used 125 photographs of door scenes as materials. The stimuli were selected from the same resources as in Experiment 2. There were 95 photographs from five functional categories, each in one dominant colour: (1) domestic ordinary—yellow; (2) garage—red; (3) church—brown; (4) business premise—green; (5) beach hut—blue. From each subset, 12 items were used as targets, 6 as related distractors, and 1 as a buffer. Thirty photographs, used at test as unrelated distractors, belonged to other categories (e.g. public, barn) and were multicoloured, pink or wooden.

#### Design and procedure

The study was built in *OpenSesame* (Mathôt et al., 2011), and conducted online on the Jatos platform. At study, participants were asked to remember door scenes, and they were informed that these door scenes belong to the following categories: domestic ordinary, garage, church, business premise, and beach hut. The study list consisted of 65 pictures (5 sets of 12 exemplars plus two buffers at the beginning and three at the end) presented in random order for 2000 ms each, followed by a blank slide presented for 300 ms.

The test lists consisted of 60 targets (12 items from each of the 5 categories), 30 related distractors (six items from the same five categories), and 30 unrelated distractors (belonging to various categories, different than the five categories used for targets and related distractors). Following the full CR paradigm, the participants were informed that their task was to answer “yes” or “no” to the question that will be presented under the test picture on a particular slide. There were three types of probe questions: (a) *Was this picture of the door scene presented?* (b) *Was this picture of the door scene not presented but is similar to the presented pictures?* and (c) *Was this picture presented or is similar to the presented pictures?* At test, slides were presented in random order, at a self-paced rate. The colour/grey-scale conditions were manipulated between-subjects.

A post-hoc sensitivity power analysis (conducted using G*Power 3, Faul, et al., 2007) showed that the test for differences between parameters in the colour and grey-scale conditions had a satisfactory power of 1 − β = 0.80 to detect a small effect size *w* = 0.04, given an alpha level of 0.05, *df* = 1, and the total number of 5040 observations gathered in this experiment.

## Results and discussion

### Results based on descriptive measures

**Fig. 8:**
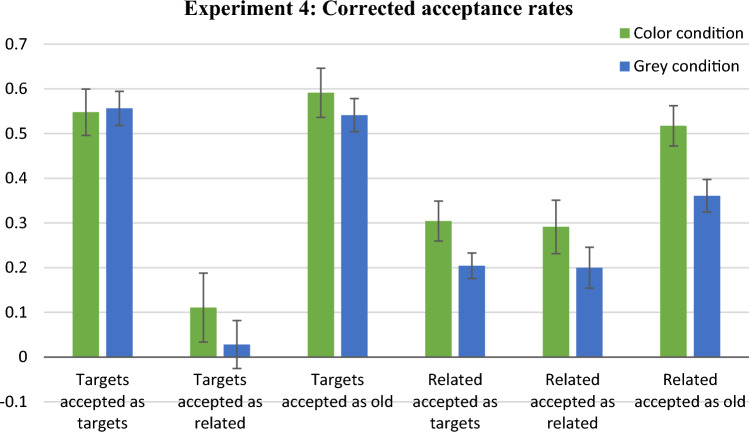
Mean proportions of response types in Experiment 4 using door scenes. Bars show the proportions of acceptances of test probes for targets or related distractors, minus the proportions of acceptances of the same probes for unrelated distractors. Error bars represent standard errors

Figure 8 presents the corrected-for-guessing mean acceptance rates for all probe-response types. One significant difference between conditions was found. Namely, the corrected acceptance rate for related distractors in the TorR? probes was significantly higher in the colour condition (*M* = 0.52, SD = 0.22) than in the grey-scale condition (*M* = 0.36, SD = 0.17), Mann–Whitney *U* = 155.00, *z* = 2.44, *p* < 0.02. When comparing false acceptances of related distractors in the T? probes, the acceptance rate was non-significantly higher in the colour condition (*M* = 0.30, SD = 0.21) than in the grey-scale condition (*M* = 0.20, SD = 0.14), Mann–Whitney *U* = 194.50, *z* = 1.56. The Bayes factor (BF_10_ = 0.846) was inconclusive, nearly equally supporting the null and the alternative hypotheses.

### Results based on multinomial modelling

The parameter estimates of the multinomial model are presented in the middle part of Table 2. The semantic familiarity parameter for related distractors was significantly higher in the colour condition than in the grey-scale condition. This result is compatible with the higher gist trace retrieval in the colour condition than the grey-scale condition found in Experiment 2. However, in contrast with Experiment 2, we found no difference between conditions in phantom recollection contribution to the false recognition of related distractors. Again, this difference may be due to the differences between CR models and their response formats. The lack of a significant difference between *P*_r_ parameters in Experiment 4 is unlikely to be explained by insufficient power, since the number of observations was much higher in Experiment 4 than Experiment 2 (5040 vs. 2940, respectively).

## Experiment 5: False memory of abstract shapes in the full CR model: follow-up experiment

Experiment 5 was conducted to further examine potential explanations for the inconsistent findings between Experiment 1 and Experiment 3. In Experiment 1, the phantom recollection parameter of the simplified CR model in the colour condition was significantly higher than that in the grey-scale condition. However, Experiment 3 did not find any differences in phantom recollection or familiarity parameters of the full CR model between conditions. The reasons for these inconsistencies may be twofold.

First, it is possible that the processes measured by one model are not identical to the corresponding processes measured by another model. This difference may be due to different response formats or the different structure of the multinomial processing trees postulated in these models. Second, there were several subtle procedural differences between these two experiments which were not intended to, but could, affect the pattern of results. Namely, in Experiment 3 the study list was shorter, there was no filler task before the test phase, and the test list was longer—it included both prototypes and non-presented list items as related distractors. To control for the potential effects of these differences, in Experiment 5, we used the same procedure as in Experiment 1. As a result, if the results of Experiment 5 are similar to those of Experiment 1, this will indicate that differences between Experiments 1 and 3 were due to subtle differences in procedure. However, if after mimicking the procedure of Experiment 1, the results of Experiment 5 are similar to the findings of Experiment 3, this will suggest that the measurement model was critical.

### Methods

#### Participants

Seventy-two participants (13 males, median age 22 years) were recruited mainly from Cardinal Stefan Wyszyński University in Warsaw. They received gift vouchers to a popular supermarket for their participation in the experiment. Participants were randomly assigned to the colour condition (*N* = 36) or the grey-scale condition (*N* = 36).

#### Design and procedure

Materials, design, and procedure were identical to those in Experiment 1 where results showed a significant difference in the phantom recollection parameter between conditions. However, at the test phase we used the response format from the full CR model. This experiment was carried out at a university lab using E-Prime 2.0.

A post-hoc sensitivity power analysis (conducted using G*Power 3, Faul, et al., 2007) showed that the test for difference between parameters in the colour and grey-scale conditions had a satisfactory power of 1 − β = 0.80 to detect a small effect size w = 0.07, given an alpha level of 0.05, *df* = 1, and the total number of 1728 observations gathered in this experiment, which is similar to Experiment 1.

## Results and discussion

### Results based on descriptive measures

**Fig. 9:**
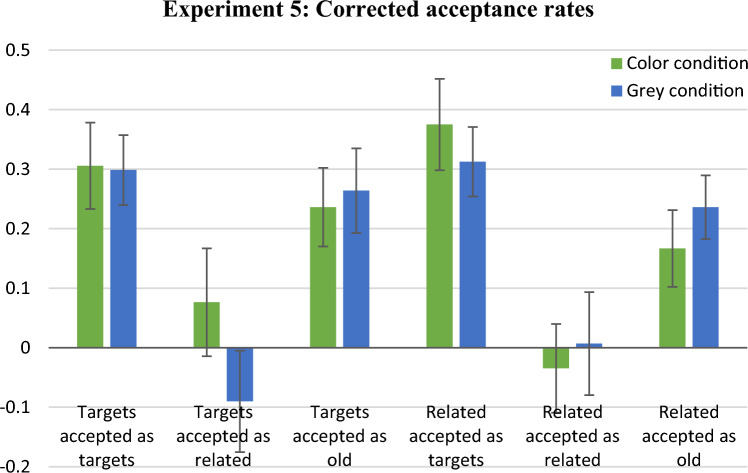
Mean proportions of response types in Experiment 5 with abstract shapes as materials. Bars show the proportions of acceptances of test probes for targets or related distractors, minus the proportions of acceptances of the same probes for unrelated distractors. Error bars represent standard errors

Figure 9 presents the corrected-for-guessing mean acceptance rates for all probe-response types. As in Experiment 3, there were no statistically significant differences between conditions. The Bayesian Mann–Whitney *U* tests yielded weak or moderate evidence for the null hypotheses, 0.244 < BF_10_ < 0.524. In particular, when comparing false acceptances of the related distractors in T? probes, the Bayes factor (BF_10_ = 0.291) indicated moderate evidence for the hypothesis that the acceptance rates for the colour and the grey-scale conditions are equal.

Since participants often guessed that unrecognised items were similar to presented items (*M* = 0.53, SD = 0.31, for both conditions together), corrected-for-guessing attributions to the “similar” category proved to be very low, suggesting that accepting targets or related items as “similar” was based more on guessing than on memory processes. The lack of differences for these two response categories must be treated with caution due to the possibility of a floor effect.

### Results based on multinomial modelling

The parameter estimates of the full CR multinomial model are presented in the bottom part of Table 2. As in Experiment 3, we found that the parameters representing semantic familiarity for targets or related distractors cannot be equated to zero for any condition, Δ*G*^2^(1)s > 8.45, *p*s < 0.004. Again, no significant difference was detected for the model parameters between the colour and grey-scale conditions. In particular, no difference was found between phantom recollection parameters. After constraining the erroneous recollection rejection parameters and recollection rejection parameters to zero in both conditions—the parameters that are absent or reduced in the simplified CR model—the model goodness-of-fit was still satisfactory, *G*^2^(4) = 2.871, *p* = 0.580. When using such a model as a baseline model, the result pattern did not change—both phantom recollection, Δ*G*^*2*^(1) = 0.07, and semantic familiarity parameters, Δ*G*^*2*^(1) = 0.16, were not significantly different between the colour and grey-scale conditions. In conclusion, it seems that the response format rather than parameter structure was responsible for the differences in the results of Experiments 1 and 5.

## General discussion

In this study, we explored the processes contributing to the false recognition of novel abstract shapes versus pictures of real objects. In five experiments, we used the conjoint recognition paradigm to assess whether gist trace retrieval contributes only to false acceptances of semantically related pictures or if it also underlies false recognition of non-semantically related abstract shapes, the latter being inconsistent with FTT’s formulation of gist representation as meaning based. The second, related aim of our experiments was to investigate the role of adding a surface feature to members of a category for false recognition, and to explore which processes in the conjoint recognition model are responsible for this effect, depending on the type of material used. In this section, we will first consider the issue of the processes underlying abstract shape recognition, then, we will discuss the effect of adding colour to targets and distractors belonging to one category. Next, we will provide some examples of alternative interpretations of our results from perspectives other than FTT and dual-recollection theory. Finally, we will discuss the possible consequences of using different versions of the CR model and paradigm for the effects observed in our experiments.

### The nature of gist representation underlying recognition of non-semantically related pictures

Fuzzy trace theory provides a well-documented and established opponent-process explanation of false memory for semantically related materials (e.g. Reyna et al., 2016). It assumes that the false acceptance of semantically related distractors is supported by the retrieval of gist memories, while it is suppressed by the retrieval of verbatim memories. The closer in meaning the targets and related distractors are, the more probable it is that distractor presentation will elicit gist memory. In DRM-like paradigms, manipulations that increase gist strength increase the incidence of false recognition of semantically related distractors, even if their associative strength is held constant (Brainerd et al., 2020). Extending this approach to account for the false memory of non-semantically related distractors implies that false acceptance can be attributed to the failure of verbatim suppression process but not to gist retrieval (Chang & Brainerd, 2021).

In the five experiments presented in the current article, we found a significant contribution of processes interpreted as gist-based to false recognition of semantically (and perceptually) related concrete distractors and perceptually (but not semantically) related abstract distractors. In particular, in Experiment 1, the gist trace retrieval parameter of the simplified CR model, was significantly higher than zero for the recognition of abstract shapes. In a similar vein, using the same abstract materials, in Experiments 3 and 5, the familiarity parameters of the full CR model also indicated a significant contribution of gist memory to the false recognition of non-semantically related distractors. At the same time, the contribution of the recollection rejection parameter to memory performance was found to be negligible (Experiments 1 and 5) or significantly higher than zero but relatively small (Experiment 3).

These results appear to be inconsistent with the FTT assumption that gist traces encode semantic relations, but instead they support the view that gist can be represented at both perceptual and conceptual levels (Naspi et al., 2021). This view is present implicitly or explicitly is some studies on false memory. For example, Arndt (2010), in his research on the effects of testing critical distractors in a font matching the font of their studied associates, explicitly assumed that fonts can produce gist representations of their commonalities when they are repeatedly presented during a study phase. In similar vein, Koustaal et al. (1999) used novel shapes without pre-existing semantic representations in their studies on false memory in amnesiacs or older adults, and assumed that false memory is based on “perceptual gist”. Oliva (2005) also used the term perceptual gist and argued that it encompasses all levels of visual information, including low-level features (such as colour). Moreover, studies using functional magnetic resonance imaging have suggested that false recognitions can be elicited by perceptual relations, since the erroneous acceptance of visually similar distractors is accompanied by enhanced activity in early visual processing regions (e.g. Bowman et al., 2019). Old-hits and old-misses evoked similar levels of activity within early visual processing regions also in studies that used novel objects or patterns without pre-existing semantic representations, as those applied in our experiments (Slotnick & Schacter, 2004; cf. Naspi et al., 2021). Moreover, it seems that the neural underpinnings of conceptual and perceptual false recognition are at least partially distinct (Garoff-Eaton et al., 2007).

In view of the aforementioned, we advocate for a reconsideration of the nature of representation underlying gist in FTT. The results of the current study and our previous research with orthographically related words (Nieznański et al., 2019; cf. Holliday & Weekes, 2006) suggest that the informational content of the relations shared by sets of items that is encoded in the gist trace is probably not limited to semantic content but also represents perceptual patterns. This concept is not consistent with the current version of the FTT (Chang & Brainerd, 2021), but it seems congruent with a description of gist traces that can be found in early FTT formulations (Brainerd & Reyna, 1990), where both senses and patterns (presumably also perceptual patterns) were included as the basis of gist representation.

Moreover, it is worth considering whether it is better to favour the view that there is a general gist representation that encompasses both perceptual patterns and semantic content, or to distinguish separate perceptual-gist and semantic-gist. The rationale for the latter account can be derived from the results of Experiment 1, in which blocked presentation did not support perceptual-gist encoding, while this form of presentation usually supports semantic-gist encoding. Arguments for separate representations may also be derived from the fact that phonological and semantic relatedness often produce dissociative effects of experimental manipulations on false memories (see: Chang & Brainerd, 2021; Tse et al., 2011). For example, evidence for the relative independence of processes underlying semantic and phonological false memories can be found in studies manipulating the presentation rate of DRM lists (Ballardini et al., 2008) or studies indicating over-additive false memory effects for hybrid lists of semantically and phonologically related words (e.g. Finley et al., 2017; Nieznański et al., 2019; Watson et al., 2001, 2003). The differences in underlying representations for the phonologic versus semantic DRM-illusions are also supported by the individual-differences study by Ballou and Sommers (2008), which found no significant correlations between false recall and recognition of critical lures across phonologically and semantically related lists.

Our interpretation of the contribution of perceptual gist to the recognition of abstract shapes is hinged on the assumption that the novel abstract objects are not processed semantically. However, it is possible that participants tried to code these non-linguistic materials in a linguistic manner. Although the abstract categories were designed to be novel and not conceptually meaningful, participants were likely able to assign conceptual labels (cf. Koutstaal et al., 2003; Pidgeon & Morcom, 2014). Thus, the role of semantic processing in the formation of gist representations for novel abstract shapes cannot be ruled out, but we consider it unlikely that semantic processing could be responsible for the entire contribution of gist to the false memory of these shapes. This issue needs further investigation.

### Representation of surface information shared by semantic or perceptual category and its contribution to false memory

The key experimental manipulation in our experiments was adding a specific colour to items in each category. We expected that related distractors that have this additional feature in common with targets would be falsely recognised more often (cf. Nieznański & Obidziński, 2022). Following Chen et al.’s (2018) suggestion that the surface details connected to a category are stored with traces of semantic information, we predicted that adding such a feature will help subjects to extract semantic information connected with the category and will increase false recognition based on gist-trace retrieval for semantically related distractors. It can be assumed that for real objects such as the door scenes, colour may be used to interpret the meaning of a scene (e.g. the brown colour of the door may somehow help identify it as a church door). However, when properties such as colour are unrelated to the meaning or pattern of an abstract shape, gist-related processes may not be used (cf. Oliva, 2005). Therefore, we tentatively expected that an increase in false recognition is more likely to occur when targets and distractors belong to a common semantic category than when the category is built on surface resemblance.

Consistent with these expectations, in the experiments using door scenes (Experiments 2 and 4), we found a significant increase in the contribution of gist-based processes to the false recognition of related distractors in the colour condition. However, in experiments using abstract shapes, the results suggested a lack of effect. More specifically, in Experiment 1, we only found an increase in phantom recollection in the colour condition, and this effect was not confirmed in follow-up Experiments 3 and 5.

The phenomenon of binding contextual (surface) features with gist representation manifests in the observation of false attributions of critical distractors to their corresponding-list’s context in studies using the DRM paradigm (Chen et al., 2018; cf. Brainerd et al., 2014). For example, in Hicks and Hancook (2002), DRM lists were presented by male and female speakers, where one speaker presented a list half of higher backward associative strength (BAS) to the critical distractor, whereas another source read half of the weak BAS. Critical lures were more often attributed to speakers who read items of a strong BAS, that is, items that were more likely to form a gist shared by the critical distractor (see also: Franks et al., 2016; Hicks & Starns, 2006; Nieznański et al., 2018). Similarly, Arndt and Reder (2003) observed that false recognition levels increased when all studied words from a given theme (gist) were presented in the same font style than when each word was associated with a single font. The surface details of the font style seemed to be connected with the stored representation of gist, which is consistent with the interpretation we proposed for our results.

An alternative possibility, derived from the recent concept of a contextual trace separate from the verbatim and gist traces (Brainerd et al., 2022a), is that the context recollection process contributes to an increase in false recognition for distractors that have an additional contextual feature (colour) in common with the targets. This explanation is only supported by our experiments using the simplified CR model, as they demonstrated a higher contribution of phantom recollection (which is the process corresponding to context recollection) in the colour condition than in the grey-scale condition. However, all three experiments using the full CR model found no differences between conditions in the parameters based on context trace retrieval (i.e. the erroneous recollection rejection or phantom recollection parameters). These inconsistencies in results between experiments depending on the model used are discussed in the next section.

Finally, let us consider the connections between the main questions raised in this article, namely, the hypotheses regarding the role of gist in the recognition of perceptually similar items and the consequences of adding a perceptual feature for false recognition of similar items. On the one hand, our finding that adding a surface feature increases the contribution of gist retrieval challenges the FTT assumption that gist is solely semantic in nature. On the other hand, this observation applied only to meaningful but not abstract objects, which seems somewhat problematic for the view that perceptual patterns are represented in the gist trace. If we assume that shared features are encoded in the gist (or perceptual gist) trace, there is no reason why adding one more common feature should not affect the gist contribution and require the mediation of semantic content. One explanation could be that our study did not reject the null hypothesis of no effect on gist contribution due to insufficient statistical power.

### Alternative theoretical perspectives

Our results showing that false memory for perceptually similar abstract shapes is driven by retrieval of representations of shared features, could be interpreted from perspectives other than fuzzy-trace theory. For example, Johnson et al. (1993) in source-monitoring theory postulated that memory accuracy (e.g. distinguishing memories of real and imagined events) depends on the availability and quality of memory characteristics in combination with the correctness of judgement processes. Both perceptual and semantic details are among the memory characteristics involved in source monitoring, therefore, both perceptual and semantic similarity can increase source misattribution (e.g. Johnson et al., 1988; cf. Bayen et al., 1996). Consistent with our approach, the effects of characteristics similarity on false recognition will be interpreted in source-monitoring theory as resulting from the availability or quality of memory records, rather than the efficiency of judgement processes. When testing perceptually or semantically related distractors, information from the target representation sometimes becomes activated and is misattributed to the distractor, leading to false acceptance (Henkel & Franklin, 1998; Henkel et al., 1998; Lyle & Johnson, 2006).

Regarding the effect of adding a feature to the studied items on the false recognition of a related distractor that shares that added feature, models of recognition would generally predict an increase in false acceptances but not necessarily different for abstract and concrete materials. Take, for example, global matching models (cf. Arndt, 2010, 2015; Hicks & Starns, 2006) which assume that memories are encoded as collections of features, including both item and context information. In these models, false recognition is a function of the match between the related distractor used as a "memory probe" during retrieval and the memory traces of all studied items. The comparison of the memory probe with all encoded items generates the stronger the activation, the higher the summed similarity of the features. For related distractors that are similar to multiple targets, recognition errors arise from summing many relatively small matches (Arndt, 2010, 2015; Arndt & Hirshman, 1998; Hicks & Starns, 2006). This approach does not differentiate between perceptual and semantic features, both can contribute to the memory probe’s global match to encoded traces. Adding any feature that is common to the memory probe and a subset of memory traces should increase global similarity and acceptance probability. Consistent with this prediction, we found a higher false acceptance rate when a perceptual feature was added to concrete pictures, but adversely we did not find this effect for abstract shapes. One could try to explain the different results for these two types of stimuli following Kinnell and Dennis (2012) by the difference in the ease with which the stimuli can be uniquely represented. In their experiments, Kinnell and Dennis (2012) found that pairs of words and scene pictures did not show a list length effect but fractals and unfamiliar faces did (as reflected in increased false alarms with long lists). The longer the list of these unfamiliar stimuli was presented at the study, the more difficult it was for participants to create unique representations for them. As predicted by global-matching theories, the amount of interference depends on the degree of overlap between item representations, therefore, overlapping representations generate an increase in false alarms rates. Such an interpretation could be applied to our results if we agree that adding a feature to concrete pictures (door scenes) leads to greater overlap of representations than in the case of abstract shapes.

A potentially more fruitful approach for interpreting our results can be found in the neurocognitive literature. For example, Cowell et al. (2019) point out that concepts of memory processes, such as recollection and familiarity, conflate neuro-computational operations (such as pattern completion or memory strength signal generation) with neural representations (which vary in properties such as dimensionality). Understanding memory mechanisms requires separating memory processes into their operations and representations. According to the representational-hierarchical account (Cowell et al., 2019), a key property of a memory is dimensionality of representations, which change along a continuum, from simple visual features to objects and scenes. As dimensionality increases, the involvement of successive brain regions changes but the operations that can act on representations in these regions are the same at all levels of the hierarchy. Sadil et al. (2019) demonstrated that associative learning occurs not only as top-down processing at the highest levels of representational dimensionality but also at lower levels, as lateral processing of associations between perceptual elements. In other words, a particular brain region may house only one class of representation, but there may be many nonspecific operations occurring in it (Cowell et al., 2019). With respect to our research, it seems reasonable to consider memories of abstract shapes as lower-dimensional representations compared to high-dimensional representations of door scenes. Operations leading to false recognitions (presumably pattern-completion retrieval) can occur for both abstract shapes and pictures of real objects, as the same operations can occur at different levels of dimensionality. One can speculate that differences in the effects of adding a feature to a category between abstract and concrete materials may depend on how this manipulation modified the properties of the representation, since representational content determines the contribution of a particular brain region to memory retrieval (Ross et al., 2018).

### Consequences of using different paradigms

Our experiments revealed that the results based on the simplified CR model (Stahl & Klauer, 2009) differ from those based on the full CR model (Brainerd et al., 2022a) with the same manipulation of adding a surface feature. Specifically, for door scenes, according to the simplified CR model, the increase in false recognition of related distractors was due to a significant increase in both phantom recollection and gist retrieval (Experiment 2), but in Experiment 4 using the full CR model, results only show a significant increase in the semantic familiarity parameter for related distractors, with no difference in phantom recollection. For abstract shapes, a significant difference in the role of phantom recollection in the false recognition of related distractors was found using the simplified CR model in Experiment 1 but not in Experiments 3 and 5 that used the full CR model. For abstract shapes, not only did the results of the modelling analyses differ between experiments, but also the findings based on descriptive measures were not consistent. In particular, in Experiment 1, the proportion of “target” responses to related distractors was higher in the colour condition than in the grey-scale condition, but in Experiments 3 and 5, the proportion of acceptance of related distractors (“yes” responses) in Target? probes did not differ between conditions. This suggests that changing the response format from a multiple-choice test that requires participants to classify each item as a target, related distractor, or unrelated distractor (in the simplified CR paradigm) to a “yes–no” format with three types of probes (in the full CR paradigm) leads to different memory performance. An even more compelling difference in performance due to subtle difference in response formats was recently demonstrated by Brainerd et al., (2022b), who found differences in participants’ performance between the Old? and Not-New? probes and between the New? and Not-Old? probes, even though these probes are logically equivalent.

## Conclusions

The results of our experiments do not support Chang and Brainerd’s (2021) suggestion that the false recognition of non-semantically related distractors is due to interference with the verbatim suppression process. Instead, we found a significant contribution of gist trace retrieval in the recognition of abstract shapes which indicates a need to revise the definition of gist trace from being purely semantically grounded and instead moving to broaden (or divide) this concept to include perceptual patterns. Alternatively, the significant contribution of familiarity to the recognition of abstract shapes may be interpreted as a rationale in favour of the thesis that familiarity is based on fluency of both perceptual and sematic processing (cf. Jacoby & Dallas, 1981; Jacoby, 1991; Mandler, 1980; but see Brainerd et al., 2022a, for opposite results). The experiments also showed that adding a surface feature increases the false recognition of related distractors by enhancing the contribution of familiarity process but only for pictures of real objects. For abstract shapes, the effect of adding a surface feature on false recognition was much more elusive and was found in an experiment using one version of the CR paradigm (Experiment 1), but not the other (Experiments 3 and 5). The use of different variants of the CR model could lead to different conclusions due to differences in structures of the multinomial models and/or, more likely, due to different response formats in the testing procedures.

## Data Availability

The raw data collected in each of the experiments presented in this article are available online at: https://osf.io/kvaxt/.

## Appendix

See Tables 3 and 4.

**Table 3:** Response frequencies used for the simplified CR model in Experiments 1 and 2

Item types	Participants responses								
	Colour/random condition			Grey/random condition			Colour/blocked condition		
	Target	Related	New	Target	Related	New	Target	Related	New
Experiment 1: Shapes									
Target	102	35	25	90	40	32	71	40	33
Related distractor	99	38	25	75	54	33	68	38	38
Unrelated distractor	60	96	168	77	92	155	59	79	150
Experiment 2: Doors									
Target	337	68	15	320	76	24			
Related distractor	139	199	82	114	187	119			
Unrelated distractor	20	92	518	50	141	439

**Table 4:** Response frequencies used for the full CR model in Experiments 3, 4 and 5

*Item types*	Participants responses											
	Colour condition						Grey condition					
	Target?		Related?		Target or Related?		Target?		Related?		Target or Related?	
	Y	N	Y	N	Y	N	Y	N	Y	N	Y	N
Experiment 3: Shapes												
Target	196	137	179	154	269	64	176	148	179	145	248	76
Related distractor	155	178	183	150	226	107	139	185	185	139	215	109
Unrelated distractor	76	257	167	166	140	193	87	237	166	158	145	179
Experiment 4: Doors												
Target	269	151	203	217	343	77	276	144	169	251	343	77
Related distractor	85	125	144	66	155	55	66	144	123	87	133	77
Unrelated distractor	17	193	84	126	42	168	21	189	79	131	55	155
Experiment 5: Shapes												
Target	42	30	44	28	54	18	44	28	31	41	58	14
Related distractor	47	25	36	36	49	23	45	27	38	34	56	16
Unrelated distractor	40	104	77	67	74	70	45	99	75	69	78	66

## Footnotes

1 Brainerd et al. (2019) defined super-overdistribution as the phenomenon in which test cues in the CR paradigm are more often remembered as belonging to a single reality state (e.g. target) than as belonging to that state or another plausible state (e.g. target or related distractor). Super-overdistribution is interpreted as being produced by gist retrieval, which is consistent with the observation that blocked presentation increases super-overdistribution effect.

## Declarations

### Supplementary data

Supplementary data for this article can be found online at: https://osf.io/kvaxt/.
